# Prevalence of Deep Vein Thrombosis in Patients With Cervical Cancer

**DOI:** 10.7759/cureus.116617

**Published:** 2026-09-20

**Authors:** Krishna Kumar Yadav, G. R. V. Prasad, Bhavana Rai, Arun Sharma

**Affiliations:** 1 Obstetrics and Gynaecology, Post Graduate Institute of Medical Education and Research, Chandigarh, IND; 2 Radiotherapy, Post Graduate Institute of Medical Education and Research, Chandigarh, IND; 3 Radiodiagnosis, Postgraduate Institute of Medical Education and Research, Chandigarh, IND

**Keywords:** cervical cancer, chemotherapy, deep vein thrombosis, prevalence, radiotherapy

## Abstract

Background

Deep vein thrombosis (DVT) is a life-threatening complication that occurs in patients with cervical cancer. The purpose of the study was to determine the prevalence of DVT in patients with cervical cancer at recruitment and to assess clinically suspected DVT during treatment and up to four months after completion of primary treatment.

Methods

The study was performed at Postgraduate Institute of Medical Education and Research (PGIMER), Chandigarh. Fifty-three histopathologically proven patients with cervical cancer were taken for ultrasound (compression and Doppler) at recruitment. Patients were then followed up during treatment and until four months after completion of primary treatment to monitor for any clinical signs or symptoms of DVT.

Results

Out of 53 patients evaluated, no cases of DVT were identified among patients with cervical cancer at recruitment on compression and Doppler ultrasound, during treatment, and until four months after completion of primary treatment, based on clinical features. A negative report could result from the small number of patients studied; most were at an early stage (stage II) and may have led to a false-negative result. Additionally, none of the patients had clear cell carcinoma, and only 16 (30.18%) patients were in advanced stages (III and IV) based on radiological evaluation.

Conclusions

DVT was not observed in patients with cervical cancer during the study period. These findings suggest that the prevalence of DVT may be low in this population. However, they should be interpreted cautiously given the limited sample size and the absence of routine follow-up imaging. Larger prospective studies are needed to further evaluate the risk of DVT in patients with cervical cancer.

## Introduction

The fourth most common cancer in women, cervical cancer is estimated to result in 604,127 new cases and 341,831 deaths globally in 2020, or roughly 3.4% of all cancer-related deaths, according to Global Cancer Statistics 2020 [1]. Cervical cancer accounts for roughly 6-29% of all female cancers in India [2]. Among patients with gynecological cancer, venous thromboembolism (VTE) is the second most common cause of death [3]. VTE occurs in 3% to 25% of patients with gynecological malignancies [4]. Women with gynecological malignancies are known to be more susceptible to VTE due to factors such as thrombogenic chemotherapy, prolonged surgery, pelvic masses compressing the vasculature, and advanced age.

Notably, in patients with cervical cancer, the prognosis is poor when DVT develops alongside gynecological malignancy [5]. When VTE develops in patients with locally advanced cervical cancer, their prognosis worsens, increasing morbidity as well as mortality. The detection of DVT in patients with cervical cancer may influence clinical management, as cancer-associated VTE requires appropriate anticoagulant therapy. Low molecular weight heparin (LMWH) is considered a standard treatment option for cancer-associated VTE [5].

As several studies have reported an increased incidence of DVT during and after radiotherapy, chemotherapy, and in the postoperative period, it is essential to determine the status of DVT in cervical cancer patients before starting treatment. This study aimed to determine the prevalence of DVT in patients with histopathologically confirmed cervical cancer at recruitment and to assess the occurrence of clinically suspected DVT during treatment and up to four months after completion of primary treatment.

## Materials and methods

This was an observational analytical study conducted in the Departments of Obstetrics and Gynecology and Radiotherapy at the Postgraduate Institute of Medical Education and Research (PGIMER), Chandigarh. Patient recruitment was carried out over a period of 12 months, followed by three to four months of follow-up until completion of primary treatment. Patients with histopathologically confirmed cervical cancer who were untreated at the time of recruitment were included. Patients were excluded if they had another co-existing malignancy, had used oral contraceptive pills or hormone replacement therapy within one month before recruitment, had known protein C or protein S deficiency or Factor V Leiden mutation, had received chemotherapy before recruitment, or were unwilling to provide informed consent.

Sample size

A total of 53 patients with histopathologically confirmed cervical cancer were enrolled. The sample size was determined by a statistician based on the planned study duration and the limited patient recruitment during the COVID-19 pandemic.

Recruitment

Patients were recruited from the Department of Obstetrics and Gynecology and the Department of Radiotherapy. Recruitment was initiated after obtaining approval from the Institutional Ethics Committee. This observational analytical study was conducted in the Department of Obstetrics and Gynecology, PGIMER, Chandigarh, in collaboration with the Departments of Radiodiagnosis and Radiotherapy.

Details of scanning

Patients with cervical cancer attending the gynecology outpatient department (OPD) or radiotherapy OPD who fulfilled the eligibility criteria and provided prior informed written consent were taken to the ultrasound room for DVT scanning by a trained radiologist. Ultrasound examination was performed using a Philips IU22 system equipped with a 5-15 MHz linear array transducer or a 1-5 MHz curved transducer (Philips Ultrasound, Inc., Bothell, USA), with settings adjusted for venous vascular studies, including a low pulse repetition frequency (PRF) color Doppler setting and a low wall filter.

Using the transducer, scanning was performed from the common femoral vein to the calf veins of both lower limbs. Gray-scale imaging with manual compression of the venous lumen using the transducer was performed first, followed by color Doppler evaluation. Features suggestive of deep vein thrombosis included a non-compressible venous segment on manual compression and absence of venous flow on color Doppler imaging. A positive finding on either compression ultrasonography or color Doppler examination was considered a DVT-positive scan.

Clinical follow-up method

Patients were informed of the signs and symptoms of DVT at the time of recruitment and were advised to contact the investigator if they developed any such symptoms during treatment or follow-up. Patients were also contacted by the investigator during the follow-up period; however, there was no fixed or standardized interval for clinical assessment. No patient reported signs or symptoms suggestive of DVT during the follow-up period.

Statistical analysis

Data were entered into Microsoft Excel (Microsoft Corporation, Redmond, USA), and statistical analysis was performed using SPSS version 22.0 software (IBM Corporation, Armonk, USA). Continuous variables were assessed for normality using the Shapiro-Wilk test. Normally distributed continuous variables were expressed as mean ± standard deviation (SD), while non-normally distributed variables were presented as median and interquartile range (IQR). Categorical variables were summarized as frequencies and percentages. Data were presented in tables. Since no patient developed deep vein thrombosis (0/53), comparative analyses and multivariable logistic regression to identify predictors of DVT were not performed because there were no outcome events available for analysis. 

Ethical justification

The study was conducted in accordance with the ethical principles of the Indian Council of Medical Research (ICMR) and the Declaration of Helsinki (2013). Ethical approval was obtained from the Ethics Committee of PGIMER, Chandigarh, before initiation of the study. Written informed consent was obtained from all participants after explaining the purpose, procedures, potential risks, and benefits of the study. Participant privacy and confidentiality were maintained, and participants were informed of their right to withdraw from the study at any time without affecting their care. The study involved no additional radiation exposure, and all measures were taken to minimize potential risks to the participants.

## Results

Using compression and color Doppler ultrasonography, 53 patients with cervical cancer were evaluated for DVT. None of the 53 patients had deep vein thrombosis at the time of recruitment. The DVT assessment at recruitment is presented in Table 1.

**Table 1:** DVT assessment at recruitment (n = 53) DVT: deep vein thrombosis.

Category	Variables	Number of subjects (n = 53)	Percent of subjects
DVT Status	Yes	0	0%
	No	53	100%
	Total	53	100%

All 53 patients, after DVT was ruled out at recruitment on ultrasound scan, were followed during treatment and up to four months after completion of primary treatment for signs and symptoms of DVT. None of the patients developed any features of DVT during the follow-up period. The clinical assessment findings for DVT during and after treatment are presented in Table 2.

**Table 2:** Clinical assessment of DVT during and after treatment (n = 53) DVT: deep vein thrombosis.

Category	Variables	Number of subjects (n = 53)	Percent of subjects
Signs and symptoms of DVT on recruitment	Signs and symptoms present	0	0%
	Signs and symptoms are absent	53	100%
	Total	53	100%
Signs and symptoms of DVT during and after treatment	Signs and symptoms present	0	0%
	Signs and symptoms are absent	53	100%
	Total	53	100%

The distribution of different variables, including demographic characteristics, comorbidities, tumor characteristics, anthropometric and lifestyle factors, and treatment-related factors, is presented in the following tables.

Age and parity status

Patients in our study ranged in age from 28 to 86, with a mean age of 53.96 ± 11.8 years. However, we did not find any cases of DVT. Out of 53 patients, 0 (0%) were nulliparous, two (3.77%) were primiparous, and 51 (96.23%) were multiparous (≥2 para). None of the patients was unmarried. The age and parity distribution of the study participants is presented in Table 3.

**Table 3:** Distribution of study participants by age and parity (n = 53)

Category	Variables	Number of subjects (n = 53)	Percent of subjects
Age in years	≤ 30	1	1.88%
	31-40	5	9.43%
	41-50	16	30.18%
	51-60	15	28.30%
	61-70	13	24.52%
	71-80	2	3.77%
	≥ 81	1	1.88%
	Total	53	100%
Parity	Nulliparous	0	0%
	Primiparous	2	3.77%
	Parity 2-4	45	84.90%
	Parity ≥5	6	11.32%
	Total	53	100%

Body mass index (BMI), comorbid Illnesses, and smoking

The mean BMI of the study's subjects was 24.4 ± 4.0 kg/m², with a maximum of 34.7 and a minimum of 14.2. Three (5.66%) patients were underweight, whereas the majority, around 29 (54.71%) patients, were of normal weight. None of the 15 (28.30%) overweight or 6 (11.32%) obese patients experienced VTE. In our study, twelve patients (22.64%) had hypertension, which was the most common comorbid illness, followed by two patients (3.77%) with diabetes mellitus and one patient (1.88%) with hyperlipidemia. Three patients (5.66%) had multiple comorbid illnesses. Out of 53 patients, fifty-one (96.22%) were non-smokers, and two patients (3.77%) were smokers. The distribution of participants according to BMI, comorbidities, and smoking history is presented in Table 4.

**Table 4:** Distribution of participants by BMI, comorbidities, and smoking history (n = 53) BMI: body mass index, CAD: coronary artery disease.

Category	Variables	Number of subjects (n = 53)	Percent of subjects
BMI in kg/m^2^	< 18.5 (underweight)	3	5.66%
	18.5-24.9 (normal weight)	29	54.71%
	25-29.9 (overweight)	15	28.30%
	> 30 (obesity)	6	11.32%
	Total	53	100%
Comorbid illness	Hypertension	12	22.64%
	Diabetes mellitus	2	3.77%
	Hyperlipidemia	1	1.88%
	CAD	0	0%
	> 1 Comorbid illness	3	5.66%
	None	35	66.03%
	Total	53	100%
History of Smoking	Yes	2	3.77%
	No	51	96.22%
	Total	53	100%

Contraception and human papillomavirus (HPV) vaccination

Only 8 (15.09%) patients in our study used barrier methods inconsistently, and none had received the HPV vaccine. The distribution of study participants according to recent contraceptive use and HPV vaccination history is presented in Table 5.

**Table 5:** Distribution of study participants according to recent contraceptive use and HPV vaccination history (n = 53) HPV: human papillomavirus, OCP: oral contraceptive pills, Cu-T: Copper-T.

Category	Variables	Number of subjects (n = 53)	Percent of subjects
Contraception used in the past month(up to one month before recruitment)	None	43	81.13%
	Barrier	8	15.09%
	OCP	1	1.88%
	Cu-T	1	1.88%
	Total	53	100%
History of HPV vaccination	Vaccinated	0	0%
	Not vaccinated	53	100%
	Total	53	100%

Histological type and grade

The most prevalent form of cervical cancer in our study, squamous cell carcinoma, was observed in 51 patients (96.22%). Adenocarcinoma was found in one patient (1.88%), and another patient (1.88%) had small-cell neuroendocrine carcinoma. However, none of the patients had clear cell carcinoma, and none developed VTE.

Of the 53 patients in our study, four (7.54%) had grade 1 disease, while 34 (64.15%) had grade 2 disease. Ten patients (18.86%) had grade 3 disease, which is associated with a poor prognosis. Grading was unavailable for five patients (9.43%). None of the patients developed VTE. The histological type and grade of the study participants are presented in Table 6.

**Table 6:** Distribution of study participants according to histological type and grade (n = 53)

Category	Variables	Number of subjects (n = 53)	Percent of subjects
Histological type	Squamous cell carcinoma	51	96.22%
	Adenocarcinoma	1	1.88%
	Other (Small cell neuroendocrine carcinoma)	1	1.88%
	Total	53	100%
Histological grade	1	4	7.54%
	2	34	64.15%
	3	10	18.86%
	Not documented	5	9.43%
	Total	53	100%

Tumor size (clinical and radiological measurements)

Out of 53 patients with cervical cancer, the majority, i.e., 39 patients (73.58%), had a tumor size > 4 cm at the time of diagnosis. Thirteen patients (24.52%) had tumors 2-4 cm in size, while only one patient (1.88%) had a tumor < 2 cm in maximum dimension. The mean tumor size was 4.77 cm.

CT/MRI was performed in all patients to measure tumor size. It was found that the majority, i.e., 26 patients (49.05%), had tumors larger than 4 cm in maximum dimension. Seventeen patients (32.07%) had a tumor size between ≥ 2 cm and < 4 cm. However, none of the patients had a tumor size < 2 cm. The mean tumor size on imaging was 4.79 cm. The distribution of study participants according to clinical and radiological tumor size is presented in Table 7.

**Table 7:** Distribution of study participants according to clinical and radiological tumor size (n = 53)

Category	Variables	Number of subjects (n = 53)	Percentage of subjects
Tumor size (clinical measurement)	< 2 cm	1	1.88%
	2-4 cm	13	24.52%
	> 4 cm	39	73.58%
	Total	53	100%
Tumor size (radiological measurement)	< 2 cm	0	0%
	≥ 2 cm to < 4 cm	17	32.07%
	≥ 4 cm	26	49.05%
	Not documented	10	18.86%
	Total	53	100%

Parametrial involvement (clinical and radiological assessment)

Fifty patients (94.33%) had parametrial involvement, while three patients (5.66%) did not. Parametrial involvement was assessed clinically through per rectal and per vaginal examination.

The majority of patients, 41 (77.35%), had parametrial involvement, whereas 5 (9.43%) did not. Parametrial involvement was assessed radiologically with CT and MRI. Another five patients (9.43%) showed parametrial fat stranding, which was also considered as parametrial involvement. The clinical and radiological assessment of parametrial involvement among the study participants is presented in Table 8.

**Table 8:** Distribution of participants by clinical and radiological parametrial involvement (n = 53)

Category	Variables	Number of subjects (n = 53)	Percentage of subjects
Parametrium involvement (clinical assessment)	Involved	50	94.33%
	Not Involved	3	5.66%
	Total	53	100%
Parametrium involvement (radiological assessment)	Involved	41	77.35%
	Not involved	5	9.43%
	Not documented	2	3.77%
	Others (Parametrial fat stranding)	5	9.43%
	Total	53	100%

Vaginal wall (clinical and radiological assessment) and lymph node involvement

Out of 53 patients, 31 patients (58.49%) had involvement of the upper two-thirds of the vaginal wall, and two patients (3.77%) had involvement of the lower one-third. Twenty patients (37.73%) did not have vaginal wall involvement. Vaginal wall involvement was assessed clinically via per vaginal (P/V) and per speculum (P/S) examination.

Radiologically, 32 patients (60.37%) had involvement of the upper two-thirds of the vaginal wall, and no patients had involvement of the lower one-third. Seven patients (13.20%) showed no vaginal wall involvement on imaging. Vaginal wall involvement was assessed using CT and MRI scans.

Based on imaging (CT and MRI), 44 patients (83.01%) had no lymph node involvement. Eight patients (15.09%) had lymph node involvement below the para-aortic region, and only one patient (1.88%) had para-aortic lymph node involvement. The majority of patients were lymph node-free.

Of the nine patients with lymph node involvement, seven (7/53, 13.20%) had stage III disease, and two (2/53, 3.77%) had stage IV disease based on radiological findings. The clinical and radiological assessment of vaginal wall and lymph node involvement among the study participants is presented in Table 9.

**Table 9:** Clinical and radiological assessment of vaginal wall and lymph node involvement (n = 53)

Category	Variables	Number of subjects (n = 53)	Percentage of subjects
Vaginal wall involvement (clinical assessment)	Involved, Upper 2/3rd	31	58.49%
	Involved, Lower 1/3rd	2	3.77%
	Not involved	20	37.73%
	Total	53	100%
Vaginal wall involvement (radiological assessment)	Involved, Upper 2/3rd	32	60.37%
	Involved, Lower 1/3rd	0	0%
	Not involved	7	13.20%
	Not documented	14	26.41%
	Total	53	100%
Lymph node involvement	Free	44	83.01%
	Para-aortic and above	1	1.88%
	Below para-aortic	8	15.09%
	Total	53	100%

International Federation of Gynecology and Obstetrics (FIGO) staging and treatment modalities

Based on radiological findings, 37 patients (69.81%) out of the 53 patients in our study had stage II cervical cancer. No patient had stage I disease on radiological assessment. Thirteen patients (24.52%) had stage III disease, and three patients (5.66%) had stage IV disease based on imaging. However, two (3.77%) patients were classified as stage I on clinical assessment.

We included all patients with stage I-IV disease based on clinical and radiological assessment, the majority being in stage II and III, yet none developed DVT.

We had only one (1.88%) patient who underwent surgery for stage IB disease, with a duration of three hours and approximately 500 mL of blood loss; however, she did not develop DVT. However, FIGO staging was upgraded after the histopathological examination (HPE) report and was treated with chemoradiation later. The clinical FIGO stage, radiological findings, and treatment modalities among the study participants are presented in Table 10.

**Table 10:** Distribution of study participants by clinical FIGO stage, radiological findings, and treatment modalities (n = 53) FIGO: International Federation of Gynecology and Obstetrics.

Category	Variables	Number of subjects (n = 53)	Percentage of subjects
Clinical FIGO stage	I	2	3.77%
	II	42	79.24%
	III	9	16.98%
	IV	0	0 %
	Total	53	100%
Radiological findings (FIGO stage)	I	0	0%
	II	37	69.81%
	III	13	24.52%
	IV	3	5.66%
	Total	53	100%
Treatment modalities	Chemoradiation only	52	98.11%
	Surgery f/b Chemoradiation	1	1.88%
	Surgery only	0	0%
	Chemotherapy	0	0%
	Total	53	100%

## Discussion

Age and parity status

According to a study by Razak et al., individuals aged 85 and older had an incidence rate more than 10 times higher (6.96 per 1,000 person-years) than those aged 45-54 years (0.72 per 1,000 person-years) [6]. A systematic review and meta-analysis of case-control studies indicates a positive association between high parity and cervical cancer [7]. 

BMI, comorbid illnesses, and smoking

Obese women are more likely than women of normal weight to develop cervical cancer, per a systematic review and meta-analysis [8]. Patients with medical comorbidities such as hypertension, diabetes, hyperlipidemia, and coronary artery disease had a higher five-year incidence of VTE, according to a study that examined data from 2003 to 2008 in the National Health Insurance Database, provided by Taiwan's National Health Research Institutes [9].

Contraception and HPV vaccination

Since cervical cancer is mainly associated with HPV infection, the barrier method of contraception is considered a protective factor against its development. The systematic review found that the HPV vaccine works best against disease outcomes linked to HPV when administered at younger ages [10].

Histological type and grade

According to a study done at the University of Tsukuba Hospital between November 2004 and December 2017, patients with adenocarcinoma had a higher incidence of VTE than patients with squamous cell carcinoma (8.6% vs. 7.2%) [11]. In a 2019 prospective cross-sectional descriptive study by Marliyya et al., conducted at Ahmadu Bello University Teaching Hospital among 67 patients with cervical cancer, all cases of DVT occurred in cervical squamous cell carcinoma patients [12].

A study that was not specifically about cervical cancer found that patients with high-grade tumors were much more likely to develop VTE than those with low-grade tumors (hazard ratio, 2.0; 95% CI, 1.1 to 3.5; P =.015) [13].

Tumor size (clinical and radiological measurements)

A study found that the risk of VTE with cervical cancer increased ninefold for tumors larger than 50 mm (10.2% in >50 mm vs. 1.2% in <50 mm) [4]. According to Satoh et al., tumors exceeding 50 mm may serve as an independent risk factor for VTE [14]. Patients with cervical cancer in advanced stages (III+IV), tumor size >4 cm, clear cell carcinoma, lymph node involvement, and extrauterine tumor spread have a higher chance of developing DVT [11].

Vaginal wall (clinical and radiological assessment) and lymph node involvement

An independent and significant risk factor for pretreatment VTE was identified in a study conducted at the University of Tsukuba Hospital between November 2004 and December 2017: lymph node (LN) enlargement, which indicates metastasis and tumor spread beyond the primary organ [11].

FIGO staging and treatment modalities

We included all patients with stage I-IV disease based on clinical and radiological assessment, the majority being in stage II and III, yet none developed DVT. A study at the University of Tsukuba Hospital from November 2004 to December 2017 found that tumor spread outside of the primary organ, stage III/IV advanced malignancy, and LN enlargement, suggesting metastasis, were independent and significant risk factors for pretreatment VTE [11]. They found that stage III/IV disease had a 12.2% incidence of DVT compared with 4.0% in stage I/II, and stage IV had the highest incidence (24.6%) [11].

We had only one (1.88%) patient who underwent surgery for stage IB disease, with a duration of 3 hours and approximately 500 mL of blood loss. In a 2022 study by Hongle et al., perioperative VTE occurred in 28 (8.3%) of 338 patients. While 18 patients (5.5%) experienced postoperative VTE, ten events (3.0%) happened before surgery. In the same study, out of 18 patients who developed postoperative VTE, ten patients had a surgery duration of >3 hours [15]. 

A 2016 study concluded that advanced-stage disease is independently associated with an elevated risk of VTE compared to early-stage disease, with a 2-year cumulative risk of distant metastatic disease at 44.8% [hazard ratio (HR) 4.13, 95% confidence interval (CI) 1.06-10.7, P = 0.003] and locally-advanced disease at 13.4% (HR 2.46, 95% CI 1.17-4.43, P = 0.004), in contrast to stage IA1-IB1 at 4.1%. Moreover, chemotherapy treatment (HR 2.46, 95% CI 1.30-4.66, P = 0.006) was identified as an independent risk factor correlated with an elevated risk of VTE [16].

The variables, including demographic characteristics, comorbidities, tumor characteristics, anthropometric and lifestyle factors, and treatment-related factors, were categorized and analyzed. A total of 56 participants were recruited for the study, satisfying the inclusion criteria. Of the 56 subjects recruited, 3 (5.35%) were excluded after recruitment due to loss to follow-up during the early period, before the first cycle of chemotherapy and radiation was initiated. The remaining 53 patients with cervical cancer were screened for DVT using ultrasound (compression and color Doppler). Compression ultrasonography and color Doppler were performed in all 53 patients.

In India, cervical cancer is the second most common gynecological cancer and continues to be a major cause of morbidity and mortality [17]. Cervical cancer incidence and mortality rates are 13.1 and 6.9 per 100,000 people worldwide, respectively, whereas they are 14.7 and 9.2 per 100,000 in India [17]. Deep vein thrombosis (DVT) is reported in association with cervical cancer.

From the literature, the incidence of DVT in gynecological cancer widely varies from 3-25% (4) and 0-34% [9] in cervical cancer. Treatment modalities such as radiotherapy, chemotherapy, and surgery are also considered to increase the incidence of clinical VTE [18], which may be silent and potentially fatal. It is generally known that women with gynecologic malignancies are more likely to develop VTE due to increased risk factors, including cancer diagnosis, advanced age, pelvic masses that strain the vasculature, extensive surgery, and thrombogenic chemotherapy [19]. Hence, this study was conducted to diagnose VTE before initiating primary treatment. This is because DVT can independently increase morbidity and mortality in cancer patients, so its pretreatment diagnosis in patients with cervical cancer may alter the primary treatment strategy. 

Limitations of the study

The study has some limitations, including a small sample size and non-consecutive recruitment. Patients with advanced disease, adenocarcinoma, neuroendocrine carcinoma, and surgically treated patients were few in number. Clear cell carcinoma was not identified in any patient during the study period. Follow-up ultrasonography (USG) was not performed in asymptomatic patients, so asymptomatic DVT may have been missed.

## Conclusions

In this study of patients with cervical cancer, DVT was not observed during the study period. These findings suggest that the prevalence of DVT may be low in this population; however, they should be interpreted cautiously given the limited sample size and the absence of routine follow-up imaging. Larger prospective studies are needed to further evaluate the risk of DVT in patients with cervical cancer, particularly across different disease stages and treatment settings.
